# Increased Prolonged Sitting in Patients with Rheumatoid Arthritis during the COVID-19 Pandemic: A Within-Subjects, Accelerometer-Based Study

**DOI:** 10.3390/ijerph20053944

**Published:** 2023-02-23

**Authors:** Ana Jessica Pinto, Diego Rezende, Sofia Mendes Sieczkowska, Kamila Meireles, Karina Bonfiglioli, Ana Cristina de Medeiros Ribeiro, Eloisa Bonfá, Neville Owen, David W. Dunstan, Hamilton Roschel, Bruno Gualano

**Affiliations:** 1 Applied Physiology and Nutrition Research Group, Laboratory of Assessment and Conditioning in Rheumatology, Hospital das Clínicas HCFMUSP, Faculdade de Medicina FMUSP, Universidade de Sao Paulo, Av. Dr. Arnaldo, 455, Sao Paulo 01246-903, Brazil; 2 Rheumatology Division, Faculdade de Medicina FMUSP, Universidade de Sao Paulo, Av. Dr. Arnaldo, 455, Sao Paulo 01246-903, Brazil; 3 Baker Heart and Diabetes Institute, 99 Commercial Road, Melbourne, Victoria 3004, Australia; 4 Centre for Urban Transitions, Swinburne University of Technology, John St, Melbourne, Victoria 3122, Australia; 5 Institute for Physical Activity and Nutrition, School of Exercise and Nutrition Sciences, Deakin University, 1 Gheringhap Street, Geelong, Victoria 3220, Australia; 6 Food Research Center, University of Sao Paulo, R. do Lago, 250, Sao Paulo 05508-080, Brazil

**Keywords:** sedentary behavior, physical activity level, inflammatory arthritis, pain, fatigue, health-related quality of life

## Abstract

Background: Social distancing measures designed to contain the COVID-19 pandemic can restrict physical activity, a particular concern for high-risk patient groups. We assessed rheumatoid arthritis patients’ physical activity and sedentary behavior level, pain, fatigue, and health-related quality of life prior to and during the social distancing measures implemented in Sao Paulo, Brazil. Methods: Post-menopausal females diagnosed with rheumatoid arthritis were assessed before (from March 2018 to March 2020) and during (from 24 May to 7 July 2020) social distancing measures to contain COVID-19 pandemic, using a within-subjects, repeated-measure design. Physical activity and sedentary behavior were assessed using accelerometry (ActivPAL micro). Pain, fatigue, and health-related quality of life were assessed by questionnaires. Results: Mean age was 60.9 years and BMI was 29.5 Kg/m^2^. Disease activity ranged from remission to moderate activity. During social distancing, there were reductions in light-intensity activity (13.0% [−0.2 h/day, 95% CI: −0.4 to −0.04; *p* = 0.016]) and moderate-to-vigorous physical activity (38.8% [−4.5 min/day, 95% CI: −8.1 to −0.9; *p* = 0.015]), but not in standing time and sedentary time. However, time spent in prolonged bouts of sitting ≥30 min increased by 34% (1.0 h/day, 95% CI: 0.3 to 1.7; *p* = 0.006) and ≥60 min increased by 85% (1.0 h/day, 95% CI: 0.5 to 1.6). There were no changes in pain, fatigue, and health-related quality of life (all *p* > 0.050). Conclusions: Imposed social distancing measures to contain the COVID-19 outbreak were associated with decreased physical activity and increased prolonged sedentary behavior, but did not change clinical symptoms sitting among patients with rheumatoid arthritis.

## 1. Introduction

A preliminary, multinational survey reporting step counts provided by smartphones showed that social distancing measures to contain the spread of SARS-CoV-2 have induced physical inactivity (i.e., not meeting the physical activity guidelines) [1]. The onset of the coronavirus disease 2019 (COVID-19) pandemic has placed further spotlight on participation in sedentary behavior (i.e., time spent in a sitting or reclining posture with a low energy expenditure [≤1.5 METs]), with reported increases in daily sitting time from pre-pandemic levels ranging from 30 min up to 3 h in different populations [2,3].

Extensive epidemiological evidence has indicated that physical inactivity is a major risk factor for early mortality and chronic diseases, including obesity, type 2 diabetes, cardiovascular diseases, metabolic syndrome, certain type of cancers, and others [4]. Even though time spent in moderate-to-vigorous intensity physical activity has the strongest detrimental associations with health outcomes [5,6,7], similar (albeit, detrimental) relationships have been broadly observed for excessive time in sedentary behaviors [7,8,9,10,11,12,13,14,15,16]. Importantly, both total sitting time and prolonged, uninterrupted sitting time are associated with increased risk of all-cause mortality even after consideration of the influence of participation in moderate-to-vigorous intensity physical activity [7,8,17]. Moreover, the deleterious associations of sedentary behavior with cardiometabolic risk and all-cause mortality are most pronounced in those who are physically inactive [6,11,18,19,20].

Rheumatoid arthritis is a rheumatic autoimmune disease characterized by chronic inflammation, pain, and physical disability [21]. Clinical disease symptoms can include joint pain, swelling, stiffness, and deformity, fatigue, muscle weakness, and reduced physical functioning [22,23]. Patients with rheumatoid arthritis have a higher risk of morbidity and mortality from cardiovascular diseases [24]. This increased risk can be at least partially explained by the complex interplay between chronic inflammation, adverse effects of drugs, associated comorbidities (e.g., dyslipidemias, insulin resistance, hypertension), and lifestyle [25,26]. Despite physical activity being advocated as an integral part of disease standard care [27], physical inactivity and sedentary behavior are highly prevalent among patients with rheumatoid arthritis [28]. 

Physical inactivity and sedentary behavior are modifiable risk factors considered to be potential targets to prevent morbimortality in autoimmune rheumatic diseases [28,29]. Among patients with rheumatoid arthritis, sedentary behavior is associated with higher disease scores, increased pain, fatigue [30] and number of comorbidities, reduced aerobic capacity [31] and physical function [30], and poor self-efficacy [32]. Furthermore, physically inactive patients with rheumatoid arthritis exhibit higher cardiovascular risk factors (e.g., higher systolic blood pressure and homeostasis model assessment (HOMA) index, abnormal lipid profile) when compared to their physically active counterparts. 

Patients with rheumatoid arthritis have been shown to be more susceptible to COVID-19 infection [33] and, therefore, may be subjected to more restrictive measures of social distancing, potentially with significant impacts on their activity options, and, hence, on their burden of cardiovascular disease risk, the main cause of mortality in this population [26].

In this prospective study using a within-subjects design, we assessed physical activity and sedentary behavior levels using accelerometers in patients with rheumatoid arthritis prior to and during the imposed measures of social distancing to combat COVID-19 in Sao Paulo, Brazil. Additionally, we have assessed whether potential changes in physical activity and sedentary behavior levels would be associated with changes in pain, fatigue, and health-related quality of life.

## 2. Materials and Methods

### 2.1. Participants

Sixty-four patients diagnosed with rheumatoid arthritis were recruited from the Outpatient Rheumatoid Arthritis Clinic of the Clinical Hospital (School of Medicine, University of Sao Paulo) between March 2018 and March 2020 to participate in a randomized controlled trial (clinicaltrials.gov: NCT03186924). Thirty-five out of 64 patients with rheumatoid arthritis accepted to participate in this ancillary study.

Post-menopausal female patients diagnosed with rheumatoid arthritis, according to American College of Rheumatology European League against rheumatism collaborative initiative revised criteria [34], were recruited directly from the Rheumatoid Arthritis Outpatient Clinic of the Rheumatology Division. The exclusion criteria included: (1) participation in structured exercise training programs within the last 12 months; (2) unstable drug therapy in the last 3 months prior to and during the study; (3) Health Assessment Questionnaire score >2.0 (i.e., severe to very severe physical impairment).

This trial was approved by the local ethical committee (Commission for Analysis of Research Projects, CAPPesq; protocol code: 58340316.0.0000.0068; approval number: 1.735.096). Patients signed an informed consent form before participation in the study.

### 2.2. Experimental Design

All patients with rheumatoid arthritis had been through a clinical and physical activity assessment before the official set of social distancing measures to contain the COVID-19 outbreak, adopted on the 24 of March 2020. This facilitated the unique opportunity to track physical activity levels during the pandemic in a within-subjects, repeated measure design. We then obtained a new approval from the ethics committee for collecting data during the social distancing. Three members of our staff (DR, SMS, KM) delivered the accelerometers (ActivPAL micro™, PAL Technology, Glasgow, UK) and questionnaires to the patients at home from the 24 May to 7 July. The time elapsed for data collection between baseline and during social distancing was 12.5 months (9.9, 15.2). Patients were asked if they had adhered to the social distancing measures. All but two responded affirmatively. Data were assessed with and without the two non-compliers, and results remained the same. Thus, we reported the full data in this manuscript.

### 2.3. Physical Activity Level

Physical activity level was measured using activPAL micro™ (PAL Technology, Glasgow, UK) activity-based accelerometers before and during social distancing. Patients wore the accelerometer for 7 consecutive days (24 h/day), which was fitted using tape (3M, Tegaderm^®^, adhesive tape) on the right medial front thigh, orientated with the x-axis pointing downward, y-axis horizontally to the left and z-axis horizontally forward. Data were exported and analyzed using ActivPAL3™ software, version 8.10.9.46 (PAL Technology, UK). Data was checked by an experienced researcher and also crosschecked with a sleep diary. All data were standardized to a 16-h day in order to avoid bias from differences in patients’ wear time, by the formula: (data × 16)/wear time. Data were reported as follows: time spent sitting and lying (h/day), in prolonged sitting (h/day), standing (h/day), stepping (h/day), time spent in light-intensity physical activity (step cadency <100 steps/min [35]), time spent in moderate-to-vigorous intensity physical activity (step cadence of ≥100 steps/min [35]), and number of sit to stand (i.e., breaks) in time spent in sedentary behavior.

### 2.4. Clinical Assessment

Clinical characteristics were assessed at baseline, before the set of social distancing, Disease activity was assessed by the Disease Activity Score in 28 joints (DAS28 PCR) [36] and Clinical Disease Activity Index (CDAI) [37], in which higher scores represent more severe disease activity. The Health Assessment Questionnaire (HAQ) [38], which evaluates physical functioning in eight domains of daily life, was also used; higher scores represent greater physical disability. Disease duration, presence of comorbidities (e.g., hypertension, dyslipidemia, type 2 diabetes, depression, and other rheumatic diseases), current dose of prednisone, current use of biological agents (e.g., anti-TNF, anti-IL6, anti-IL1, B-cell depleting agents, and T-cell activation inhibiters), non-biological disease-modifying anti-rheumatic drugs (e.g., methotrexate and leflunomide), and other medications (i.e., anti-inflammatory drugs, pain killers, antihypertensive drugs, antihyperlipidemic drugs, antidiabetic drugs, and anti-depressants) were obtained by reviewing medical records and interviewing patients with rheumatoid arthritis. Blood samples (~10 mL) were collected after a 12-h overnight fast for measuring the following parameters: C-reactive protein and erythrocyte sedimentation rate. Samples were collected in vacutainer tubes and subsequently analyzed at the Clinical Hospital Central Laboratory (School of Medicine, University of Sao Paulo). C-reactive protein was determined by immunoturbidimetry. Erythrocyte sedimentation rate was assessed using an automated analyzer.

Pain, fatigue, and health-related quality of life were assessed before and during social distancing. Pain was assessed by the Visual Analogic Scale [39], in which patients graded their pain using a 10-point scale; zero means no pain and 10 means severe or unbearable pain. Fatigue was assessed by the Fatigue Severity Scale [40], in which scores range from 9 to 63; lower scores indicate lower fatigue. Physical and mental health-related quality of life were assessed by the 36-Item Short Form Survey (SF-36) questionnaire [41], in which scales (physical health: physical function, role-physical, bodily pain, and general health; mental health: vitality, social function, and role-emotional) range from 0 to 100; higher scores indicate better quality of life. 

### 2.5. Statistical Analysis

Dependent variables were tested using repeated measures mixed models, with time (Before social distancing versus During social distancing) as fixed factor and participants as random factor, with a compound symmetry covariance matrix. Delta changes in all dependent variables were calculated with the following formula: delta change = data during social distancing—data before social distancing. Associations between changes in physical activity and sedentary behavior level and changes in pain, fatigue, and health-related quality of life were tested using Pearson correlation tests. 

Statistical analysis was performed in SAS 9.3 (SAS Institute Inc., Cary, NC, USA). Data are presented as mean, estimated mean difference from the repeated measures mixed models, and 95% confidence intervals (95% CI). The significance level was set at *p* ≤ 0.05.

## 3. Results

Patients’ clinical characteristics are presented in Table 1. In summary, mean age was 60.9 years (95% CI: 58.0 to 63.7) and BMI was 29.5 Kg/m^2^ (95% CI: 27.2 to 31.9). Disease activity ranged from remission to moderate activity, as assessed by DAS28 PCR and CDAI. Disability assessed by HAQ ranged from mild to severe. Mean disease duration was 18.5 years (95% CI: 14.7, 22.3). Mean C-reactive protein was 10.8 mg/dL (95% CI: 5.5 to 16.2) and erythrocyte sedimentation rate was 28.4 mm/H (95% CI: 15.7 to 41.1). Most of the patients were using disease-modifying anti-rheumatic drugs and prednisone (85.7% and 74.3%, respectively). Hypertension, dyslipidemia and type 2 diabetes were the most frequent comorbidities (51.4%, 48.6% and 34.3%, respectively). Before social distancing, mean pain was 5.0 (95% CI: 4.1 to 6.0), fatigue was 39.3 (95% CI: 33.8 to 44.8), and physical and mental health-related quality of life were 39.8 (95% CI: 33.1 to 46.5) and 62.0 (95%CI: 52.3 to 71.7), respectively.

**Figure 1:**
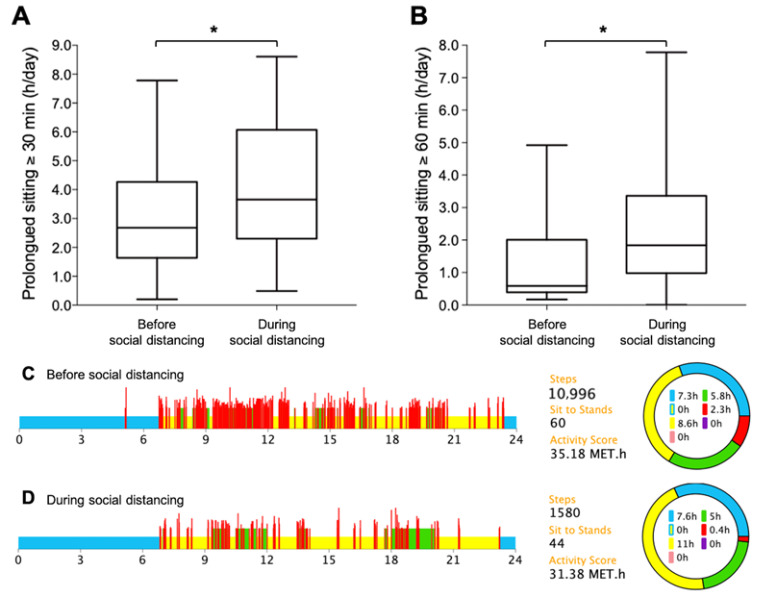
Prolonged sitting and physical activity in rheumatoid arthritis. Panels (**A**) and (**B**) depict total time spent in prolonged sitting ≥ 30 min and ≥60 min (*n* = 35). In the bottom, illustrative data showing the fall in activity and the rise in prolonged sitting in a rheumatoid arthritis patient assessed before (Panel **C**) and during the social distancing (Panel **D**). Legend: blue, sleeping time; yellow, sedentary time; green, standing time; red, stepping time (irrespective of intensity); * *p* < 0.05.

During social distancing, there were reductions in total stepping time (15.7% [−0.3 h/day, 95% CI: −0.4 to −0.1; *p* = 0.004]), in light-intensity physical activity (13.0% [−0.2 h/day, 95% CI: −0.4 to −0.04; *p* = 0.016]) and in moderate-to-vigorous physical activity (38.8% [−4.5 min/day, 95% CI: −8.1 to −0.9; *p* = 0.015]), but no changes in total standing time (−0.1 h/day, 95% CI: −0.7 to 0.5; *p* = 0.767) or total sedentary time (0.3 h/day, 95% CI: −0.4 to 1.0; *p* = 0.335) in patients with rheumatoid arthritis. However, time spent in prolonged bouts of sitting ≥ 30 min increased by 34% (1.0 h/day, 95% CI: 0.3 to 1.7; *p* = 0.006; Figure 1A) and sitting bouts ≥60 min increased by 85% (1.0 h/day, 95% CI: 0.5 to 1.6; *p* < 0.001; Figure 1B). Sit-stand transitions were reduced by 10% (−5.1/day, 95% CI: −10.3 to 0.0; *p* = 0.051). Figure 1C and Figure 1D illustrate the accelerometer data from a patient who experienced decreased activity and increased prolonged sitting after social distancing.

During social distancing, there were no changes in pain (0.31 [95% CI: −1.04 to 1.67; *p* = 0.652), fatigue (−2.3 [95% CI: −10.0 to 5.4]; *p* = 0.550), and physical and mental health-related quality of life (1.2 [95% CI: −8.2 to 10.7]; *p* = 0.796 and −9.3 [95% CI: −23.0 to 4.5], *p* = 0.183, respectively) in patients with rheumatoid arthritis.

Changes in physical activity and sedentary behavior levels were not associated with changes in pain, fatigue, and physical and mental health-related quality of life during social distancing (all *p* > 0.050).

## 4. Discussion

To our knowledge, this is the first study to track physical activity and sedentary behavior patterns before and during the COVID-19 pandemic using validated accelerometry and a within-subjects design. Our main findings suggest that social distancing (including stay-at-home order) can lead to reduced ambulatory activities and increased physical inactivity as well as increased prolonged sitting among patients with rheumatoid arthritis. In contrast, social distancing was not associated with worsened pain, fatigue, and physical and mental health-related quality of life. Physical inactivity along with too much sitting emerge as a risk factor that could be detrimental to cardiometabolic health in such a high-risk group of patients during and possibly after the COVID-19 pandemic. 

As those confined at home are less prone to perform physical activity, it has been speculated that inactivity and sedentary behavior could peak during the COVID-19 pandemic [29]. In fact, a rapid review has shown a substantial decrease in physical activity with a concomitant increase in sedentary behavior across all age groups during COVID-19 lockdown [42]. As for the Brazilian population, a national retrospective survey comprising 39,693 adults and older adults has shown a significant increase on self-reported physical inactivity and screen-based sedentary behaviors during the COVID-19 pandemic [43,44], which corroborates the objectively measured data presented herein. Such an increase in inactivity and sedentary behavior is of particular concern for those who are usually hypoactive and show higher risk of cardiovascular diseases, this being the case of patients with rheumatoid arthritis (see the patients’ comorbidities in Table 1) [26,28].

Observational and experimental evidence demonstrates that inactivity can predispose to pathological states and poor outcomes [45]. Sedentary behavior can add to the adverse impacts of physical inactivity in impairing cardiovascular health [46]. Consequently, individuals who are both physically inactive and highly sedentary are at the highest risk for poor outcomes [6,20], which might be the case for patients with autoimmune rheumatic diseases, as they commonly spent most of their daily hours engaged in sedentary behavior and did not achieve minimum levels of moderate-to-vigorous physical activity [28]. Namely in rheumatoid arthritis, the estimates of physical inactivity and sedentary behavior are comparable to those of other chronic diseases (e.g., type 2 diabetes and cardiovascular diseases), groups in which both physical inactivity and sedentary behaviors are associated with poor disease prognosis and mortality [9,10,11,13,47], as well as poor health-related outcomes (i.e., higher disease activity score, disease symptoms and number of comorbidities, and lower physical capacity and functioning) [28]. In rheumatoid arthritis, regular participation in exercise improves disease symptoms, inflammatory markers, cardiometabolic risk factors, and physical capacity [48,49]. However, regular participation in moderate-to-vigorous physical activity may not be feasible for some patients, especially those with poor mobility or during disease flares.

Interestingly, we observed that even in the absence of changes in total sedentary time, prolonged sitting time rose considerably. Prolonged, uninterrupted bouts of sedentary behavior are associated with all-cause mortality [8], whereas well-controlled studies show that very-light to light-intensity active interruptions in prolonged sedentary time (e.g., 2 min of walking for every 30 min of sitting) can elicit immediate improvements in cardiometabolic risk factors [50]. Recent evidence has shown that light-intensity physical activity is associated with lower disability, disease activity and cardiovascular risk in rheumatoid arthritis, in contrast to excessive sitting [28,51]. Additionally, a crossover randomized trial demonstrated that performing 3-min bouts of light-intensity walking every 30 min of sitting (total: 42 min) resulted in improved glycemic (i.e., glucose, insulin, and c-peptide) and inflammatory (i.e., IL-1β, IL-1ra, IL-10, and TNF-α) markers when compared to 8 h of prolonged, uninterrupted sitting in postmenopausal females with rheumatoid arthritis [52]. This raises the need for widespread recommendation of breaking-up prolonged sitting whenever possible (e.g., 3 min breaks of light-intensity walking every 30 min of sitting) to avoid poor health outcomes during the pandemic, which tend to be more restrictive for high-risk groups for COVID-19, such as those with autoimmune rheumatic diseases [33], a condition associated with lower vaccine responses, which may enforce more vulnerable patients to maintain some degree of physical distance and home isolation for as long as the pandemic endures.

Our findings suggested social distancing did not affect pain, fatigue, and physical and mental health-related quality of life. Qualitative evidence in patients with rheumatoid arthritis demonstrate that patients reported no changes in physical health outcomes. Conversely, they noted social distancing resulted in worsened mental health-related symptoms [53]. Additionally, changes in these variables did not associate with changes in physical activity and sedentary behavior. Because this study was performed 2 to 4 months after the set of social distancing measures, we cannot rule out that such a short period of exposure did not allow detecting impairments in these outcomes. Alternatively, it is possible that patients with rheumatoid arthritis may be more resilient than general population to the detrimental impacts of the pandemic on overall health.

The main strengths of this study are its within-subjects design and the use of posture-based accelerometers, which enables an objective and a comprehensive assessment of sedentary behavior patterns. The limitations include the relatively low sample size; lack of measurement of mood and use of medication and supplements during social distancing, which may also alter habitual physical activity; and the inability to stablish a cause-and-effect relationship between changes in behavior with social distancing measures, although elements of temporality and plausibility do support our assumptions.

## 5. Conclusions

Imposed social distancing measures to contain the COVID-19 outbreak were associated with decreased physical activity and increased prolonged sitting time, but no changes in clinical symptoms (pain, fatigue, and health-related quality of life) among patients with rheumatoid arthritis. Since this has the potential to increase the burden of cardiovascular diseases in such high-risk group of patients, attention to maintaining their activity levels is an urgent consideration during the pandemic, and possibly thereafter since inactivity and sedentariness may carry over as consequences of the outbreak.

## Figures and Tables

**Table 1:** Patients’ characteristics prior to the COVID-19 pandemic.

Variable	*n* = 35
Age, years	60.9 (58.0 to 63.7)
BMI, kg/m²	29.5 (27.2 to 31.9)
Disease Activity Score (DAS28 PCR)	3.4 (2.9 to 3.8)
Clinical Disease Activity Index (CDAI)	10.1 (6.7 to 13.4)
Health Assessment Questionnaire (HAQ)	1.2 (0.9 to 1.4)
C-reactive protein, mg/L	10.8 (5.5 to 16.2)
Erythrocyte sedimentation rate, mm/H	28.4 (15.7 to 41.1)
Disease duration, years	18.5 (14.7 to 22.3)
Comorbidities, *n* (%)	
Hypertension	18 (51.4%)
Dyslipidemias	17 (48.6%)
Type 2 diabetes	12 (34.3%)
Fibromyalgia	11 (31.4%)
Other rheumatic diseases	18 (51.4%)
Depression	7 (20.0%)
Medication, *n* (%)	
DMARDs	30 (85.7%)
Leflunomide	19 (54.3%)
Methotrexate	16 (45.7%)
Prednisone	26 (74.3%)
Biological agents	12 (34.3%)
Anti-inflammatory drugs	11 (31.4%)
Pain killers	20 (57.1%)
Antihypertensive drugs	18 (51.4%)
Antihyperlipidemic drugs	17 (48.6%)
Antidiabetic drugs	12 (34.3%)
Anti-depressants	14 (40.0%)

Data presented as mean (95% CI) or *n* (%). Other rheumatic diseases include osteoarthritis, osteoporosis and/or Sjogren’s syndrome. Abbreviations: DMARDs, disease-modifying antirheumatic drugs; TNF, tumor necrosis factor.

## Data Availability

Data will be made available upon reasonable request through email.
